# Signaling pathway STAT1 is strongly activated by IFN-β in the pathogenesis of osteoporosis

**DOI:** 10.1186/s40001-014-0074-4

**Published:** 2015-01-07

**Authors:** Claudine Seeliger, Lilianna Schyschka, Zienab Kronbach, Angela Wottge, Martijn van Griensven, Britt Wildemann, Helen Vester

**Affiliations:** Department of experimental Trauma Surgery, Klinikum rechts der Isar, Technical University Munich, Ismaninger Str. 22, 81675 Munich, Germany; Berlin-Brandenburger Centrum für Regenerative Therapien, Julius Wolff Institut, Charité-Universitätsmedizin Berlin, Augustenburger Platz 1, 13353 Berlin, Germany

**Keywords:** IFN-β, Osteoblasts, Osteoclasts, Osteoporosis, STAT1 signaling

## Abstract

**Background:**

Despite extensive research, the underlying pathological mechanisms of osteoporosis are not completely understood. Recent studies have indicated a distinct role for the IFN-β/STAT1 pathway in bone metabolism. An inhibitory effect of IFN-β on osteoclastogenesis has been detected and STAT1/2 has been shown to influence osteoblastic bone metabolism. So far, no data concerning the IFN-β/STAT1 pathways in osteoblasts and osteoclasts from osteoporotic and non-osteoporotic patients are available. The aim of the study was to analyze these pathways in both cell types.

**Methods:**

Osteoblasts were isolated from the femoral heads of 12 osteoporotic and 11 non-osteoporotic patients and monocytes were differentiated into osteoclasts. After the differentiation period, cells were stimulated once with 20 and 100 ng/mL IFN-β for 4 days. Viability, activity, bone metabolism-related genes, and the proteins Fra1, SOCS1, STAT1, p-STAT1, and TRAF6 were analyzed.

**Results:**

Viability, activity, and gene expressions were not affected by stimulating the osteoblasts. However, in osteoporotic osteoclasts, which display a significantly higher basal osteoclastic activity, the stimulation with IFN-β lead to significant inhibition. Further, an increased STAT1 activation was detected in both cell types with no significant differences between the groups. Regarding the phosphorylation of STAT1, no significant influence was detected in osteoblasts but the IFN-β stimulation led to a significant increase of p-STAT1 in osteoclasts of both groups.

**Conclusions:**

IFN-β is a principal mediator in the pathogenesis of osteoporosis by inhibiting osteoclasts and inducing and activating STAT1. Our results also confirm this in cells from osteoporotic and non-osteoporotic patients. Strong inhibitory effects on the osteoclastogenesis of osteoporotic osteoclasts were detectable. Nevertheless, osteoblast activity was not negatively affected by IFN-β stimulation. These results may contribute to a better understanding of the underlying pathological signaling pathways of osteoporosis.

**Electronic supplementary material:**

The online version of this article (doi:10.1186/s40001-014-0074-4) contains supplementary material, which is available to authorized users.

## Background

Osteoporosis is defined as a systemic skeletal disease characterized by a loss of bone mineral density and microarchitectural deterioration of bone tissue, with a consequent increase of bone fragility and susceptibility to fractures [1]. Typical osteoporotic fractures are localized in the hip, wrist, and/or vertebrae. It represents a global public health issue for elderly women with a very high lifetime risk of any osteoporotic fractures. In women and men, the risk range lies within 40 to 50% and 13 to 22%, respectively [2]. The underlying mechanism of osteoporosis is an imbalance between bone resorption (osteoclast activity) and bone formation (osteoblast activity). Several mechanisms, such as hormone deficiency (lack of estrogen), deficiency of calcium, disturbed vitamin D metabolism, and an imbalance in parathyroid hormone metabolism, have been suggested [3].

The loss of bone density associated with estrogen withdrawal is a result of a marked increase in osteoclast activity. It is also known that estrogen deficiency is not the sole responsible factor for increased osteoclastogenesis. Estrogen has not been found to have a major direct effect on osteoclast activity, but its withdrawal stimulates osteoclast activity indirectly [4]. One hint of how this might be achieved comes from the interactive nature of osteoclasts in the bone microenvironment. Activated osteoclasts are usually found in the presence of accessory cells, including stromal cells, cells in the osteoclast lineage, and cells involved in the inflammatory response. These cells possess the ability to express pro-inflammatory cytokines. There is now strong evidence suggesting that production of pro-inflammatory cytokines in response to estrogen withdrawal during menopause might be responsible for the characteristic loss of bone density through their effect on osteoclast activity [5]. For example, Sato et al. [6] showed an increase of interleukin (IL)-7 in ovariectomized mice, which induces osteoclast generation, but did not affect receptor activator of nuclear factor-kB (RANK) ligand (RANKL) mRNA expression in osteoblasts.

Recently, focus has been drawn on the interaction between the immune system and bone metabolism. Abnormal activation of the immune system and expression of pro-inflammatory cytokines, such as IL-6, IL-10, and tumor necrosis factor α (TNF-α), lead to bone destruction in diseases such as rheumatoid arthritis and animal models deficient in immunomodulatory molecules often develop an unexpected skeletal phenotype. Thus, the crosstalk between immune and skeletal systems or the interdisciplinary field called “osteoimmunology” has attracted much attention in recent years [5,7,8].

Consequently, the analysis of the intracellular signaling pathways of the basic pathology of osteoporosis is of high interest. In this context, studies could show an inhibitory effect of interferon β (IFN-β) on osteoclastogenesis. IFN-β is induced by RANKL, which interferes with the RANK/RANKL system resulting in the inhibition of the RANKL-induced c-FOS expression. As investigated by Takayanagi et al. [9,10], global IFN-β-knockout mice display an osteoporotic phenotype due to an increased osteoclastogenesis. As signal transducer and activator of transcription 1 (STAT1) is critically involved in IFN-β signaling pathways, some authors analyzed its importance for bone metabolism. By using Biblio-MetReS, a tool to reconstruct gene and protein networks from automated literature analysis, Sun et al. [11] could identify STAT1 as an important gene involved in osteoporosis. Furthermore, STAT1 gene expression was reported to be up-regulated in femur tissue in osteoporotic mice and human [12,13]. *In vivo* STAT1^−/−^ mice show an increased osteoclast number and enhanced osteoclastic bone resorption. Nevertheless, these mice had an increased bone mass due to excessive osteoblast differentiation overcoming bone degradation [14].

Thus far, there are no data concerning the influence of IFN-β signaling pathways, including STAT1 protein synthesis, in human osteoporotic bone cells. Therefore, the aim of the study was to analyze the effect of IFN-β on osteoblasts and osteoclasts of patients suffering from osteoporosis in comparison to healthy controls.

## Methods

Macrophage colony-stimulating factor (M-CSF), RANKL, and IFN-β were obtained from Peprotech (Hamburg, Germany). Fetal calf serum (FCS), L-glutamine, cell culture medium, trypsin/EDTA, penicillin, streptomycin, LSM 1077, and phosphate buffered saline (PBS) were purchased from PAA Laboratories GmbH (Pasching, Austria). Complete protease inhibitor was from Roche (Mannheim, Germany). Collagenase type II was obtained from Biochrom (Berlin, Germany). All other chemicals were purchased from Sigma (Munich, Germany).

### Human samples

Twelve patients suffering from an osteoporotic fracture after a minor trauma (in the following referred to as osteoporosis group) and 11 patients who underwent elective hip replacement due to arthrosis without osteoporosis (in the following referred to as non-osteoporosis group) were enrolled. Patients were recruited if the following criteria were met: femoral neck or pertrochanteric fracture, and indication of surgical treatment. Exclusion criteria were malignancy, polytrauma, benign ovarian cysts except endometrioma, inflammation, known chronic, systemic, metabolic, or endocrine diseases, including polycystic ovarian syndrome, insulin-dependent diabetes mellitus, bisphosphonate therapy, hormone therapy in the previous 3 months, and any medical history or signs of other inflammatory disease. The femur heads of all patients were collected during the implantation of the prostheses. This study was approved by the local ethical review committee of the Faculty of Medicine of the Technical University of Munich, which works in accordance with national regulations and the ICH-GCP guidelines (project number 2413/09a). The study was performed according to the declaration of Helsinki in its newest version. The patients provided informed written consent. The classification of the patients in the osteoporosis and the non-osteoporosis group was based on clinical, radiographic and DXA evaluation. Bone density was additionally evaluated via q-CT (Philips iCT, Best, the Netherlands and Mindways calibration phantom and software, Austin, TX, USA) of the femoral head obtained from the patients. The demographic data of included patients are presented in ‘Additional file 1: Table S1’.

### Isolation and culture of primary human osteoblasts

Primary human osteoblasts were isolated from femur heads of patients undergoing total hip replacement. Briefly, cancellous bone was removed mechanically from the femur head, washed five times with PBS, and digested for 1 h at 37°C with an equal volume of 0.07% Collagenase II in PBS [15]. The enzymatic reaction was stopped by osteoblast culture medium (MEM with Earle’s Salts/Ham’s F12 with L-glutamine, 10% FCS, 100 U/mL penicillin, 100 μg/mL streptomycin, 50 μM L-ascorbate-2-phosphate, and 50 μM β-glycerol-phosphate) [16,17]. Bone pieces were transferred to a 175 cm^2^ cell culture flask with 25 mL cell culture medium. The supernatant was centrifuged at 650 × *g* for 10 minutes. Afterwards, the supernatant was aspirated and the cell pellets were resuspended and distributed into flasks. The medium was changed every 4 to 5 days and after two weeks the osteoblasts were growing out of the bone pieces. The cells were expanded and used for experiments from passage 3 at a density of 2.0 × 10^4^ cells/cm^2^. All experiments were performed in triplicate.

### Generation of human osteoclasts

Buffy coats of 36 mL EDTA full blood from the osteoporotic and non-osteoporotic patients undergoing total hip replacement were isolated by density gradient centrifugation using LSM 1077 [15,18]. Afterwards, CD14 MACS Beads (Miltenyi Biotech, Germany) were used to separate monocytes from lymphocytes and 1 × 10^6^ cells per well were seeded on a 24 well plate. Monocytes were cultivated for differentiation with osteoclast culture medium (α-MEM, 10% FCS standard, 2% L-glutamine, 1% Penicillin/streptomycin, 20 pg/mL RankL, and 5 pg/mL M-CSF). Experiments started at day 10 of cultivation and were performed in triplicate.

### Stimulation of the cells with IFN-β

A sterile stock solution with the end concentration of 1 mg/mL IFN-β in water was prepared according to the manufacturer’s recommendation. For the experiments, osteoclastically-differentiated monocytes and mature osteoblasts were stimulated in triplicates for 4 days with 0, 20, and 100 ng/mL IFN-β. These concentrations and time points were evaluated by preliminary tests. After 4 days of stimulation, the viability (resazurin), osteoblast alkaline phosphatase (AP) activity, and the tartrate-resistant alkaline phosphatase (TRAP) activity of osteoclasts were analyzed. Additionally, the RNA from the cell lysates of osteoblasts and the protein from the cell lysates of both cell types were collected.

### Viability measurement of osteoblasts

Viability was determined by resazurin conversion. Briefly, 1/10 volume of a 0.025% (w/v) Alamar Blue solution (Biozol, Eching, Germany) was added to the osteoblasts after the 4 days of stimulation. After 2 h incubation at 37°C, the fluorescence was measured (excitation = 544 nm; emission = 590 nm) and corrected to the background control (solvent mixture without cells). Experiments were performed in triplicate.

### Osteoblast alkaline phosphatase (AP) assay

Ubiquitous AP present in the body is typically highly concentrated in growing bone. Therefore, AP can be used for osteoblast characterization. Moreover, it is a typical enzyme used as a marker for osteoblast identification *in vitro*. Osteoblasts plated in triplicate were washed twice with PBS. Afterwards, 500 μL p-nitrophenyl phosphate (pNPP) buffer (0.2% 4-nitrophenyl-phosphate disodium salt hexahydrate, 50 mM glycine, 1 mM MgCl_2_, 100 mM TRIS, pH 10.5) were added and the plates were incubated for 30 min at 37°C. pNPP buffer alone served as a negative control. The formation of 4-nitrophenol metabolized by alkaline phosphatase was determined in a spectrophotometer at 405 nm and calculated according to a standard curve. The signal was normalized to the relative protein content determined by sulforhodamine staining as previously reported [19].

### Semi-quantitative RT-PCR analysis of osteogenic genes

Total RNA from osteoblasts was extracted using Trizol reagent, according to the manufacturer’s recommendations (PeqLab, Erlangen, Germany). The amount and purity of RNA was determined by spectrophotometry. RNA integrity was examined by agarose gel electrophoresis. RNA was transcribed to cDNA using the First Strand cDNA Synthesis Kit (Fermentas, St. Leon-Rot, Germany). Sequences of both the forward and reverse primers and conditions used in the semi-quantitative RT-PCR are listed in ‘Additional file 1: Table S2’. Semi-quantitative RT-PCRs were performed as followed: 95°C 5 min, (35 cycles: 95°C 40 s 40 s annealing, 72°C 40 s) 72°C 10 min. The 35 cycles represent the linear phase of these PCR analyses. c-DNA from the SAOS-2 cells served as positive control and DEPC-H_2_O was used as a negative control. PCR amplicons were visualized by applying ethidium bromide in a 2% (w/v) agarose gel. For quantification, signals were analyzed with the software ImageJ 1.42q (National Institute of Health, Maryland).

### Osteoclast resorption ability

Osteoclasts, known to remove mineralized matrix by lysing organic bone, are able to form identifiable lacunas on dentin chips. For the development verification of osteoclasts, sterilized dentin chips were added to a 24-well plate onto which isolated monocytes were plated at a density of 1 × 10^6^ cells per well. After 14 days of differentiation, the dentin chips were incubated with 10 to 15% hypochlorite solution for 30 s. After washing and wiping, the dentin chips were stained with 1% toluidine blue solution in PBS for 1 s. Afterwards, the chips were intensively washed with tap water and microscopically analyzed [20].

### TRAP activity measurement of osteoclasts

TRAP activity is known as an important marker for osteoclasts; its concentration provides information about osteoclast function. TRAP activities were measured in triplicate on day 10 of differentiation and after 4 days of stimulation with IFN-β. Unstimulated cells served as controls and 150 μL of substrate buffer solution (5 mM 4-nitrophenyl-phosphate disodium salt hexahydrate in a buffer containing 100 mM sodium acetate and 50 mM sodium tartrate dibasic dehydrate, pH 5.5) were added to 50 μL of the culture supernatant. After 1 h incubation at 37°C, the reaction was stopped with 50 μL 3 M NaOH. TRAP activity was determined using a spectrophotometer at a wavelength of 405 nm, corrected to background control (culture medium without cells) and calculated with a standard curve. The signal was normalized to the protein concentration determined by the micro Lowry assay [21].

### Western blot of osteoblasts and osteoclasts

Briefly, the cells were lysed in ice-cold RIPA lysis buffer (50 mM TRIS; 250 mM NaCl; 2% Nonidet-P40; 2.5 mM EDTA; 0.1% SDS; 0.5% DOC; complete protease inhibitor; 1% phosphatase inhibitor, Na_3_VO_4_ (100 mM), PMSF (50 mM), pH = 7.2). Protein concentration was determined according to the method of Lowry [21]. Briefly, 40 μg total protein was separated by 10% SDS PAGE and transferred to nitrocellulose membranes (Roth, Karlsruhe, Germany). Antibody description and usage conditions are summarized in ‘Additional file 1: Table S3’. Membranes were incubated with the first antibody overnight at 4°C in the dark. On the next day after washing, the incubation with the second antibody followed for 2 hours at room temperature. The development of the membrane was realized via chemiluminescence reaction and X-ray film. Densitometric analysis of the signals was performed using ImageJ.

### Statistics

All experiments were performed at least three times. Results are expressed as mean ± SEM. For the comparison of more than two data sets, a one-way analysis of variance (ANOVA) with a non-parametric Kruskal-Wallis test followed by the Dunn’s multiple comparisons test were performed. For the statistical analysis of two data sets, a non-parametric unpaired two-tailed Mann-Whitney *U*-test was performed. For all statistical analyses, GraphPad Prism version 5.01 for Windows was used (GraphPad Software, San Diego, USA). *P* <0.05 was considered as the minimum level of significance.

## Results

### Viability and AP activity of osteoblasts

**Figure 1:**
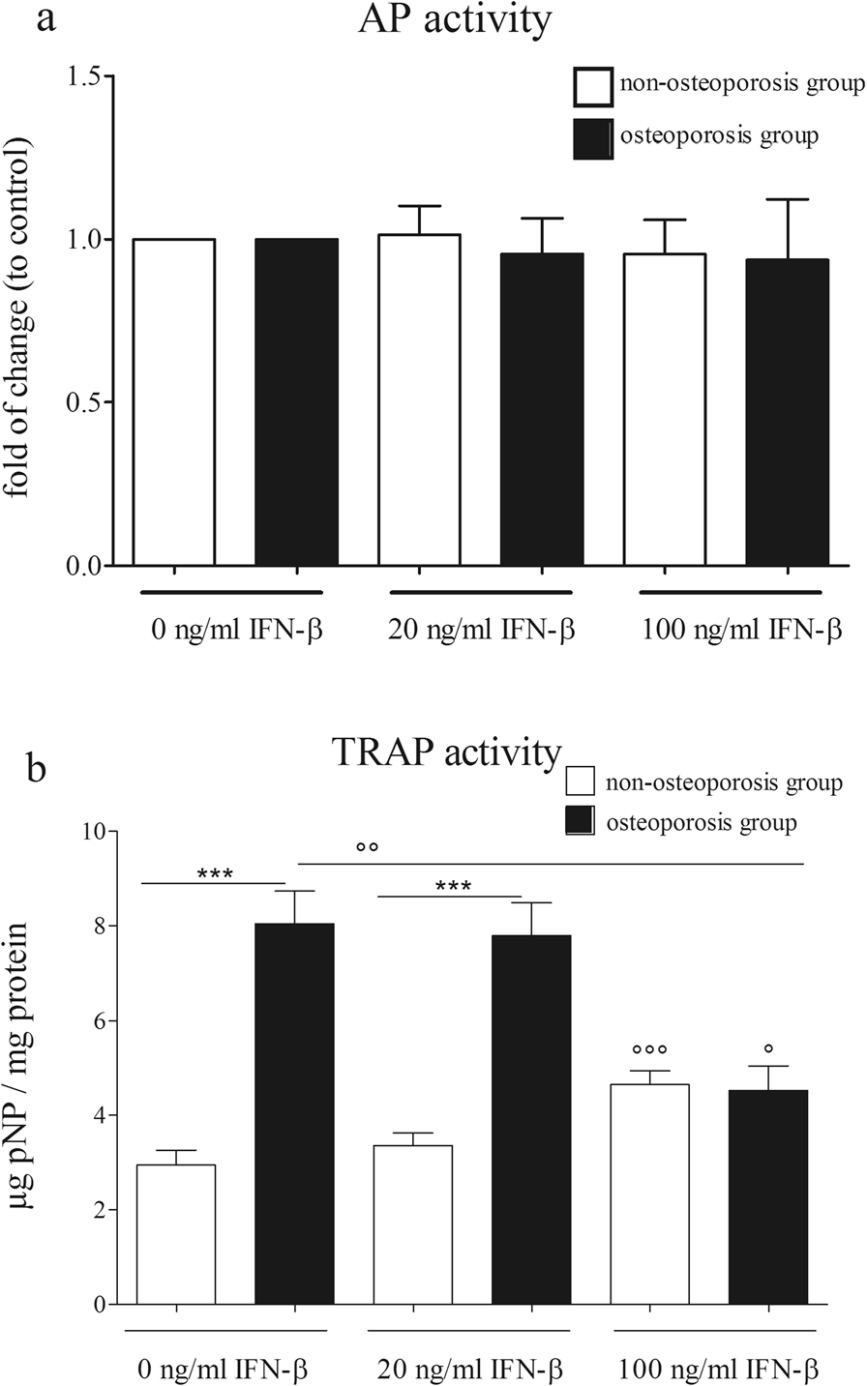
**Influence of IFN-β on the activity of osteoblasts and osteoclasts.** **(a)** IFN-β did not influence the AP activity of osteoblasts from osteoporotic and non-osteoporotic patients. **(b)** Osteoclasts of osteoporotic patients show a significantly increased TRAP-activity reducible by the stimulation with 100 ng/mL IFN-β. Bars represent mean ± sem, n = 12 osteoporosis group, n = 11 non-osteoporosis group. *** *P* <0.001 compared to the non-osteoporosis group, ° *P* <0.05, °° *P* <0.01, °°° *P* <0.001 compared to untreated cells.

Viability of osteoblasts was not influenced by stimulation with IFN-β (Additional file 1: Figure S1). Moreover, the AP activity of osteoblasts from non-osteoporotic and osteoporotic patients was not affected by IFN-β stimulation (Figure 1a).

### RNA expression in osteoblasts

IFN-β stimulation did not change the expression levels of osteocalcin (OC), osteoprotegerin (OPG), and collagen1A1 (COL1A1) in osteoblasts from both groups (Additional file 1: Figure S2).

### Resorption ability and TRAP activity of osteoclasts

Evaluated by toluidine blue staining, the multinucleated osteoclasts led to resorbed areas on the used dentin chips after the differentiation period (Additional file 1: Figure S3).

TRAP activity was measured in IFN-β-stimulated osteoclasts (Figure 1b). Osteoclasts generated from monocytes of osteoporotic patients showed a significantly (3.36 times) higher TRAP activity compared to osteoclasts from non-osteoporotic patients. By using 20 ng/mL IFN-β, the TRAP activity in osteoclasts from osteoporotic patients was significantly higher compared with cells from the non-osteoporosis group. Cells of both groups displayed significantly higher osteoclast activity by using 100 ng/mL IFN-β compared to unstimulated cells. In contrast, in the osteoporotic group, stimulation with 100 ng/mL IFN-β led to significantly (2 times) lower TRAP activity compared to the unstimulated cells.

### Synthesis or phosphorylation of IFN-β pathway-related proteins

Analysis of protein synthesis using the Western blot technique revealed comparable synthesis levels of fos-related antigen 1 (Fra1), suppressor of cytokine signaling 1 (SOCS1), and the TNF receptor-associated factor 6 (TRAF6) in the non-osteoporosis and osteoporosis groups for both cell types (Additional file 1: Figure S4).

**Figure 2:**
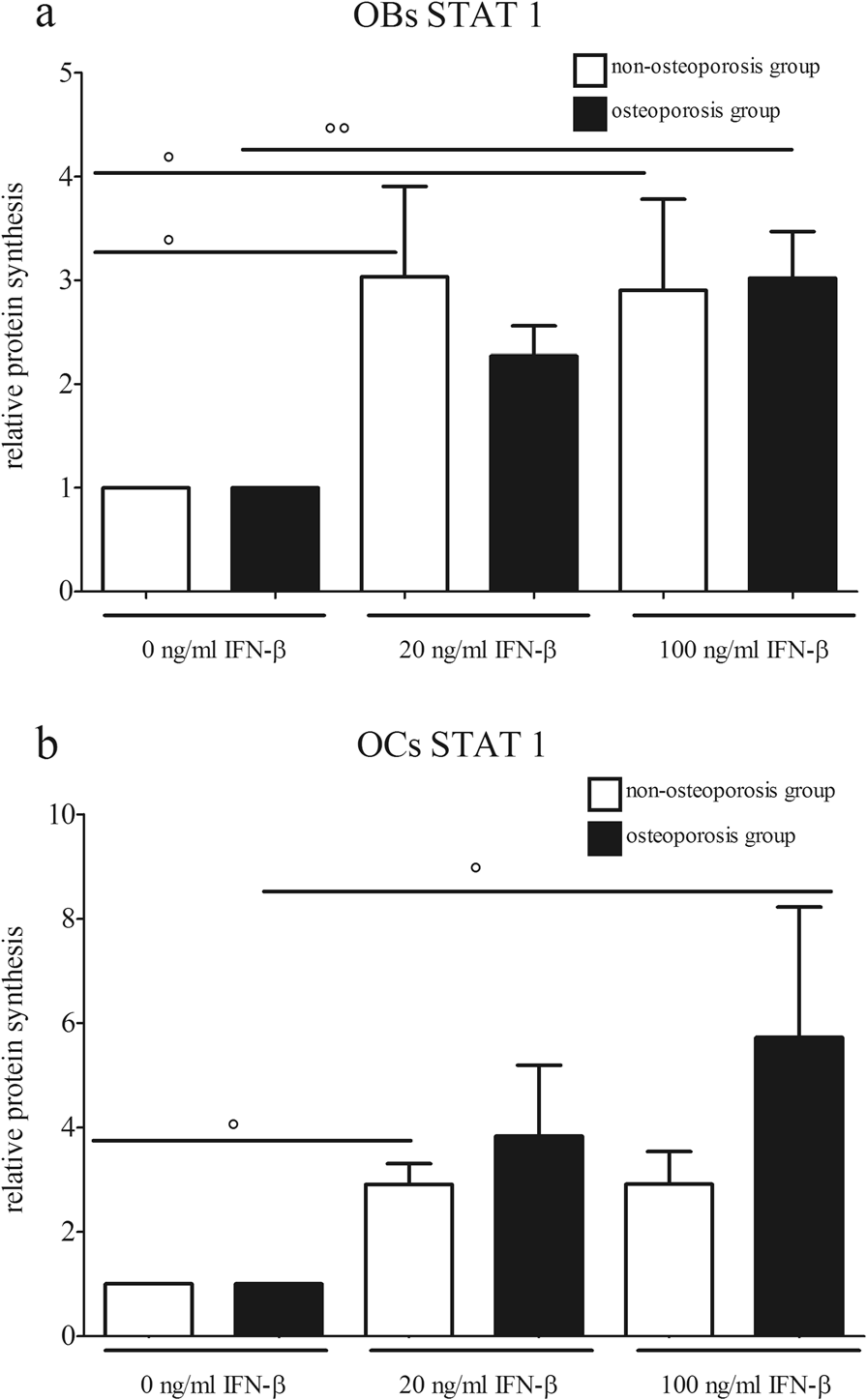
**STAT1 levels of IFN-β-stimulated cells.** STAT1 protein levels of **(a)** human osteoblasts and **(b)** human osteoclasts stimulated with IFN-β. Bars represent mean ± sem, n = 12 osteoporosis group, n = 11 non-osteoporosis group. ° *P* <0.05, °° *P* <0.01 compared to the untreated cells. OBs, Osteoblasts; OCs, Osteoclasts.

**Figure 3:**
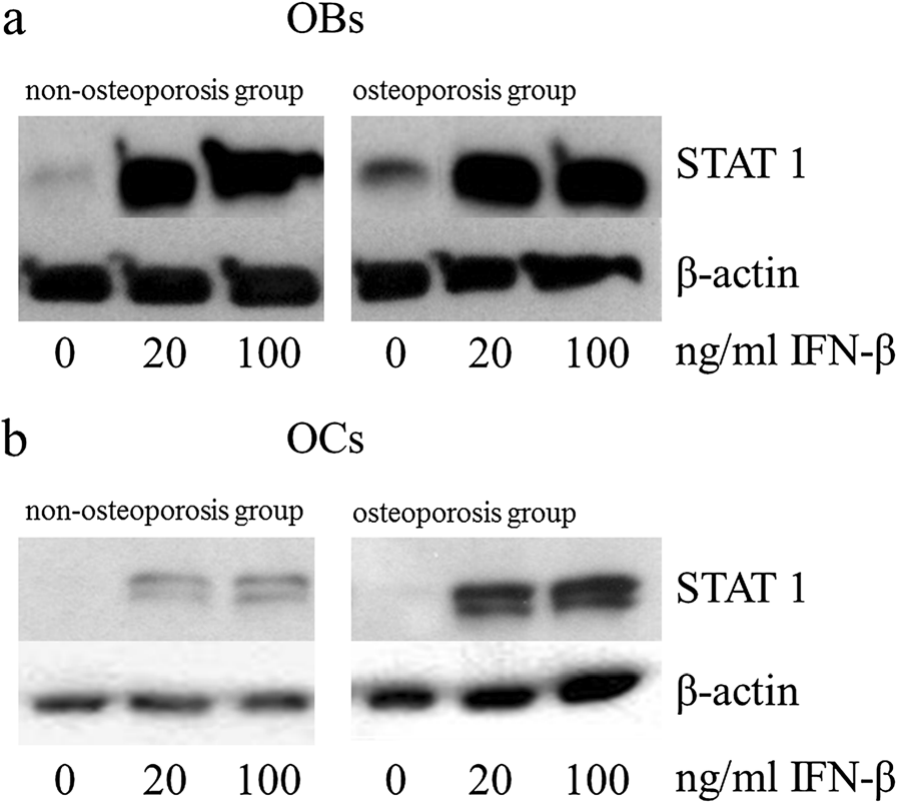
**Western blots of STAT1 in IFN-β stimulated cells.** STAT1 protein levels of **(a)** human osteoblasts and **(b)** human osteoclasts stimulated with IFN-β. Representative analyses from all experiments are shown. OBs, Osteoblasts; OCs, Osteoclasts.

**Figure 4:**
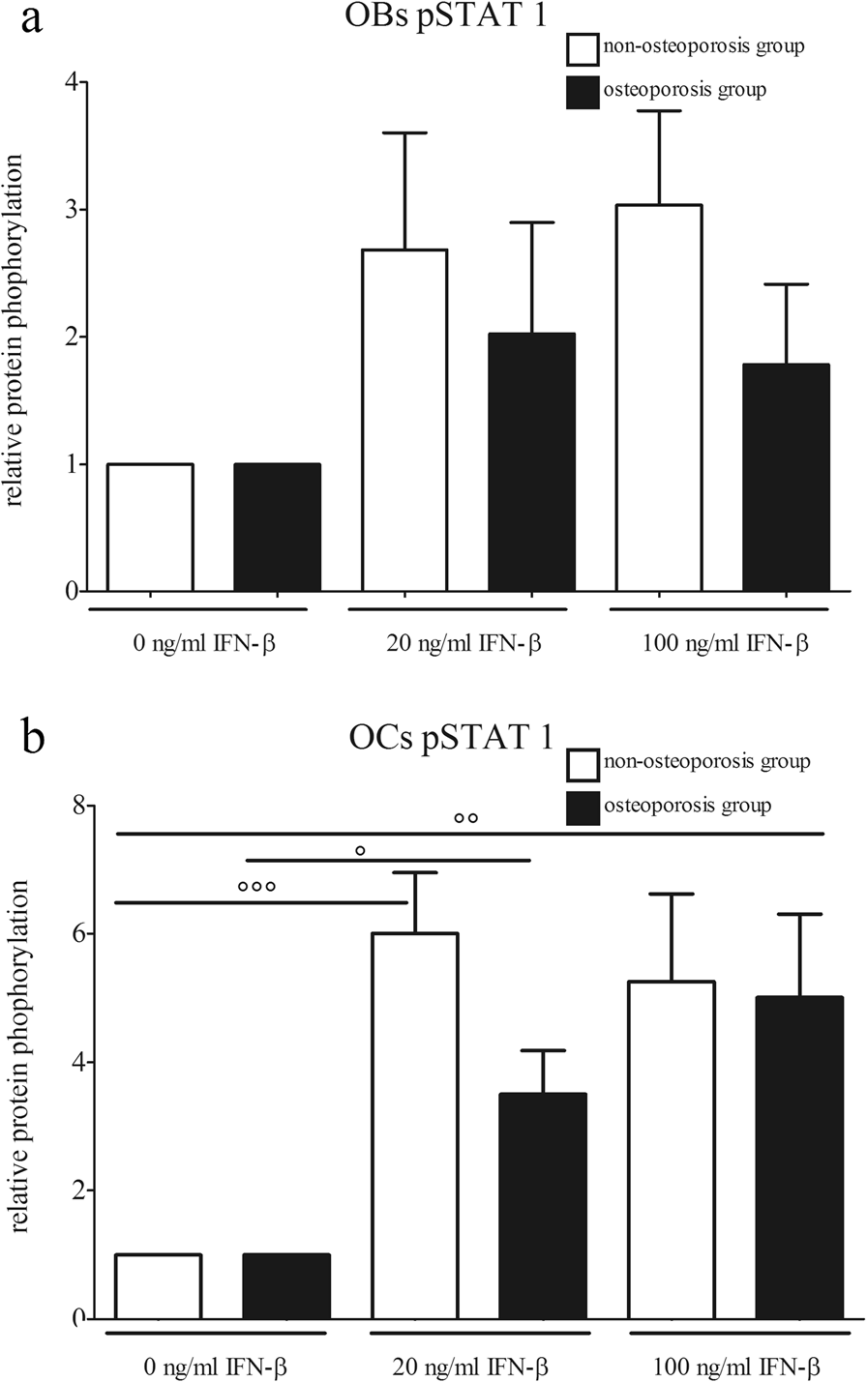
**STAT1 phosphorylation of IFN-β stimulated cells.** STAT1 phosphorylation levels of **(a)** human osteoblasts and **(b)** human osteoclasts stimulated with IFN-β. Bars represent mean ± sem, n = 12 osteoporosis group, n = 11 non-osteoporosis group. ° *P* <0.05, °° *P* <0.01, °°° *P* <0.001 compared to the untreated cells. OBs, Osteoblasts; OCs, Osteoclasts.

**Figure 5:**
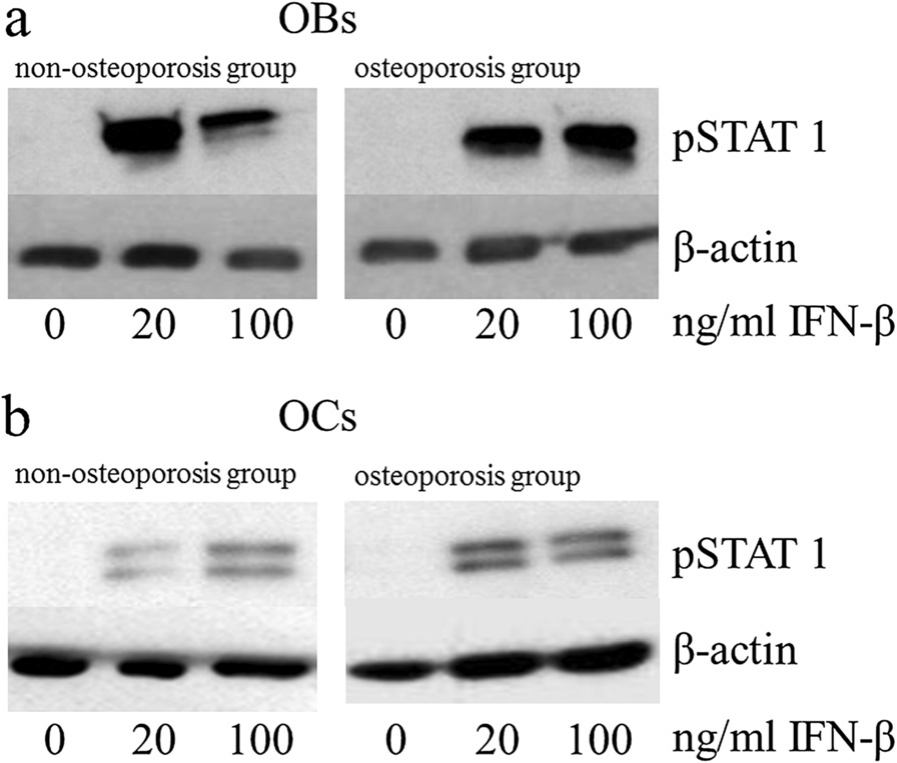
**Western Blots of STAT1 phosphorylation in IFN-β stimulated cells.** STAT1 phosphorylation levels of **(a)** human osteoblasts and **(b)** human osteoclasts stimulated with IFN-β. Representative analyses from all experiments are shown. OBs, Osteoblasts; OCs, Osteoclasts.

However, incubation with 20 and 100 ng/mL IFN-β resulted in significantly increased synthesis levels of STAT1 in osteoblasts and osteoclasts of both groups (Figure 2a, b). These expression increases were directly detectable on the corresponding Western blot films (Figure 3a, b). In particular, osteoclasts of the osteoporotic group displayed a dose-dependent increase and, after incubation with 100 ng/mL, the protein level was significantly (up to 5.7 times) higher in comparison to the unstimulated osteoclasts. Activation of the transcription factor by phosphorylation was also influenced by IFN-β, whereby, in osteoblasts, no significant change was detected between the groups (Figure 4a, b). However, phosphorylation of STAT1 in the osteoclasts was significantly increased in both groups after incubation with IFN-β. Expression changes were directly visible on the corresponding Western blot films (Figure 5a, b).

## Discussion

Natural IFN-β is produced in large quantities by fibroblasts and binds to the interferon receptor IFN-α/βR. This activates the JAK signal transducer and activator of transcription pathway to phosphorylate STAT1 and STAT2 [22,23]. Furthermore, IFN-β exerts anti-inflammatory properties through the down-regulation of IL-1β and TNF-α and enhancement of IL-10 and IL-1 receptor antagonist production [24-26]. Generally, IFN-β is used as a treatment for multiple sclerosis as some studies have shown a reduction of the relapse rate [27].

In this study, we investigated the role of IFN-β and the STAT signaling pathway in the pathogenesis of osteoporosis. IFN-β and osteoclastogenesis are induced by RANKL via induction of the c-fos gene. Binding of IFN-β to its receptor leads to an activation of the JAK/STAT signaling pathway, which results in c-fos inhibition causing a suppression of osteoclast differentiation and osteoclastogenesis [28].

As both osteoblasts and osteoclasts are involved in the pathogenesis of osteoporosis, we analyzed both cell types in osteoporotic and non-osteoporotic bones, trying to figure out the intracellular pathologies. Neither viability nor activity of osteoblasts was influenced by IFN-β stimulation. On the other hand, osteoclasts from the osteoporosis group, which displayed a high osteoclastic activity, showed a significantly reduced activity after IFN-β stimulation. This beneficial effect of IFN-β on bone metabolism by inhibition of osteoclastogenesis has been shown before and could be considered in the view of possible application approaches [10,28-30]. Our data show that osteoclast-like cells from osteoporotic patients respond differently to IFN-β stimulation with a pathological regulation of the signaling pathway. This discrepancy may due to the different basal osteoclastic activity and the lower amount of INF-β receptors on the surface of the cells. Several authors have analyzed the potential benefit of IFN-β for bone metabolism. However, contrary statements can be found in the literature. For example, Varoglu et al. [31] detected no effect of systemic application of IFN-β in patients with multiple sclerosis on bone mineral density or T-scores in the lumbal area and the femur. In contrast, Shuhaibar et al. [32] showed a positive effect of IFN-β application on bone mineral density in 37 multiple sclerosis patients receiving corticosteroid therapy. Studies have shown that stimulation with RANKL results in the induction of mRNA of IFN-β, suggesting that IFN-β is selectively involved in osteoclast regulation. The skeletal system of mice lacking IFN-β (IFN-β^−/−^ mice) exhibited severe osteopenia resulting from enhanced osteoclastogenesis [10]. This corresponds with our results of decreased TRAP activity after incubation with IFN-β.

However, in our study, no differences in protein synthesis levels of Fra1, SOCS1, and TRAF6 could be detected in cells from both groups. Moreover, incubation with IFN-β did not affect synthesis levels. This can be explained by the fact that downstream mediators, such as TRAF6 and Fra1, are induced by RANKL [28]. Further, it might have been the wrong time point for detection. Fra1, for example, is induced by c-fos, which could not be detected and which is inhibited by IFN-β-inducible genes. Moreover, SOCS1 is induced by STAT1, which was significantly increased by IFN-β stimulation. Therefore, it also might have been the wrong time point for detection of SOCS elevation.

An increase of STAT1 synthesis after IFN-β stimulation was detected in both osteoporotic and non-osteoporotic cells; however, the stimulation only led to an activation of STAT1 in osteoclasts, shown by an increased phosphorylation. In both bone cell types, the transcription factor STAT1 is activated by IFN-β and seems to participate significantly in the pathogenesis of osteoporosis. The role of STAT1 signaling in bone homeostasis was been previously described by Kim et al. [14], who detected an increased bone mass despite excessive osteoclastogenesis in STAT1-null mice. The authors suggested that, through the interaction with Runx2, STAT1 inhibits the process of bone formation *in vivo* [14].

## Conclusions

The importance of the negative regulatory effect of IFN-β in view of osteoclastogenesis has been long known, however, the differential effect on cells from osteoporotic and non-osteoporotic patients has been addressed the first time herein. Our study shows that both osteoblasts and osteoclasts are crucially involved in the pathology of osteoporosis. Further, IFN-β seems to be a major mediator acting through the induction of the important STAT1 signaling pathway.

## Additional file

Additional file 1:
**The data set supporting the results of this article are included as an additional file.** **Figure S1**: Viability of osteoblasts stimulated with IFN-β. **Figure S2**: semi-quantitative RT-PCR results of stimulated osteoblasts, osteocalcin (OC), osteoprotegerin (OPG) and collagen1A1 (COL1A1). **Figure S3**: Toluidine blue staining of on dentin chips seeded and differentiated osteoclasts. Osteoclasts remove the bone matrixes and on the dentin chip resorption-lacunas get visible. **Figure S4**: Protein levels of Fra1, SOCS1 and TRAF6 in osteoblasts and osteoclasts stimulated with IFN-β. fos-related antigen 1 (Fra1), suppressor of cytokine signaling 1 (SOCS1), TNF receptor-associated factor 6 (TRAF6), Osteoblasts (OBs), Osteoclasts (OCs). **Table S1**: Demographical characteristics of the samples. **Table S2**: Primer sequences and PCR conditions, osteocalcin (OC), osteoprotegerin (OPG) and collagen1A1 (COL1A1), glyceraldehyde 3-phosphate dehydrogenase (GAPDH).

## Abbreviations

AP: Alkaline phosphatase
FCS: Fetal calf serum
Fra1: Fos-related antigen 1
IFN-β: Interferon β
IL: Interleukin
M-CSF: Macrophage colony-stimulating factor
PBS: Phosphate buffered saline
RANK: Receptor activator of nuclear factor-kB ligand
RANKL: Receptor activator of nuclear factor-kB ligand
SOCS1: Suppressor of cytokine signaling 1
STAT1: Signal transducer and activator of transcription 1
TNF-α: Tumor necrosis factor α
TRAF6: TNF receptor-associated factor 6
TRAP: Tartrate resistant alkaline phosphatase
