# Supplementary material for: Signaling pathway STAT1 is strongly activated by IFN-β in the pathogenesis of osteoporosis
Source: Eur J Med Res. 2015 Jan 7;20(1):1. doi: 10.1186/s40001-014-0074-4 (PMC4300729; doi:10.1186/s40001-014-0074-4)
Supplement: Additional file 1: — The data set supporting the results of this article are included as an additional file. Figure S1: Viability of osteoblasts stimulated with IFN-β. Figure S2: semi-quantitative RT-PCR results of stimulated osteoblasts, osteocalcin (OC), osteoprotegerin (OPG) and collagen1A1 (COL1A1). Figure S3: Toluidine blue staining of on dentin chips seeded and differentiated osteoclasts. Osteoclasts remove the bone matrixes and on the dentin chip resorption-lacunas get visible. Figure S4: Protein levels of Fra1, SOCS1 and TRAF6 in osteoblasts and osteoclasts stimulated with IFN-β. fos-related antigen 1 (Fra1), suppressor of cytokine signaling 1 (SOCS1), TNF receptor-associated factor 6 (TRAF6), Osteoblasts (OBs), Osteoclasts (OCs). Table S1: Demographical characteristics of the samples. Table S2: Primer sequences and PCR conditions, osteocalcin (OC), osteoprotegerin (OPG) and collagen1A1 (COL1A1), glyceraldehyde 3-phosphate dehydrogenase (GAPDH). [file 40001_2014_74_MOESM1_ESM.pdf]

Signaling pathway STAT1 is strongly activated by IFN-β in the pathogenesis of osteoporosis

European J. of medical research

Additional file 1

Claudine Seeliger[1*§], Lilianna Schyschka[1*], Zienab Kronbach[2], Angela Wottge[1], Martijn van Griensven[1], Britt Wildemann[2], Helen Vester[1]

[1] Department of experimental Trauma Surgery, Klinikum rechts der Isar, Technical University Munich, Munich, Germany

[2] Berlin-Brandenburger Centrum für Regenerative Therapien, Julius Wolff Institut, Charité-Universitätsmedizin Berlin, Berlin, Germany

Corresponding author contact information:

Dr. rer. nat. Claudine Seeliger
Email: seeliger@uchir.me.tum.de

Additional figures:

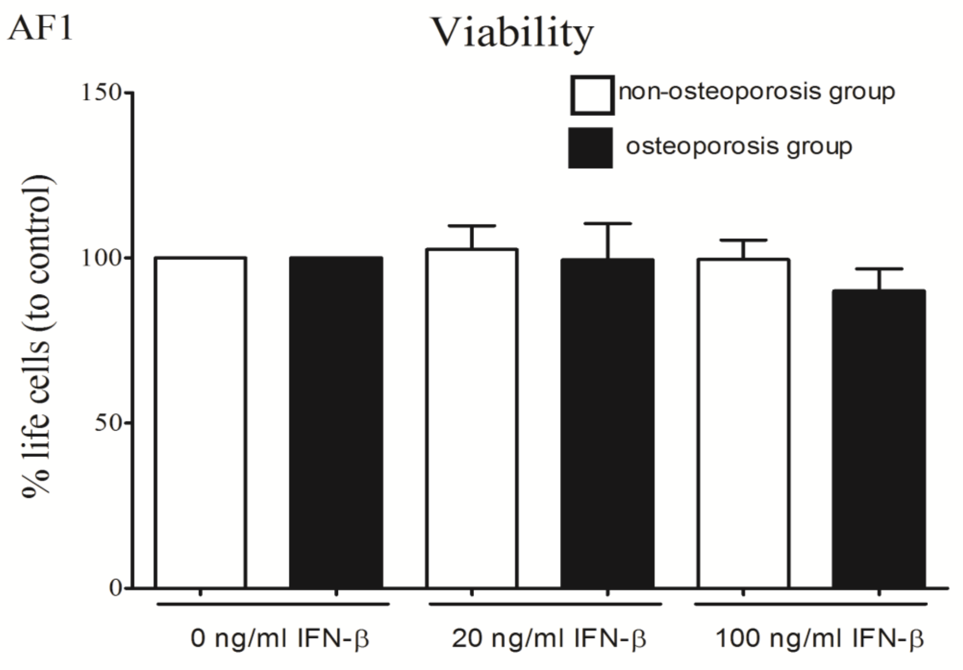

Figure AF1 - Viability of osteoblasts stimulated with IFN-β

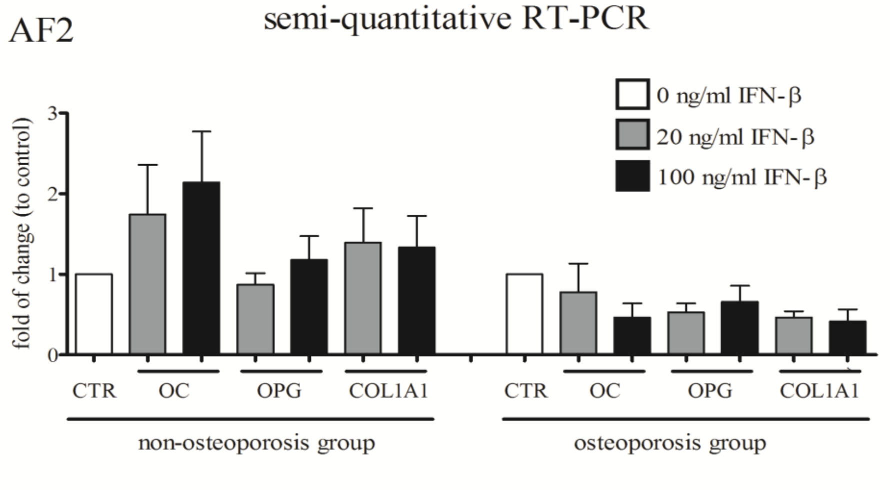

Figure AF2 - semi-quantitative RT-PCR results of stimulated osteoblasts
osteocalcin (OC), osteoprotegerin (OPG) and collagen1A1 (COL1A1)

AF3

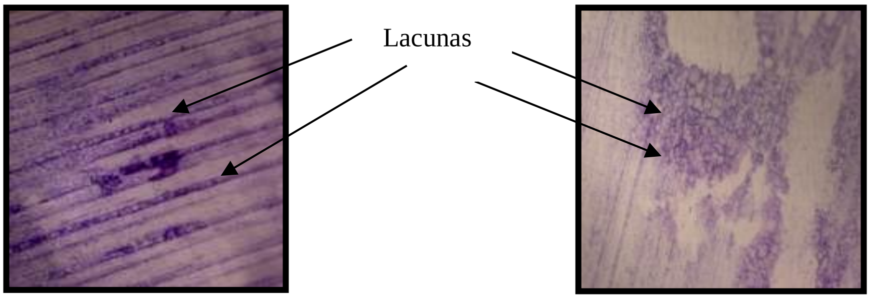

Figure AF3 - Toluidine blue staining of on dentin chips seeded and differentiated osteoclasts. Osteoclasts remove the bone matrixes and on the dentin chip resorption-lacunas get visible

AF4

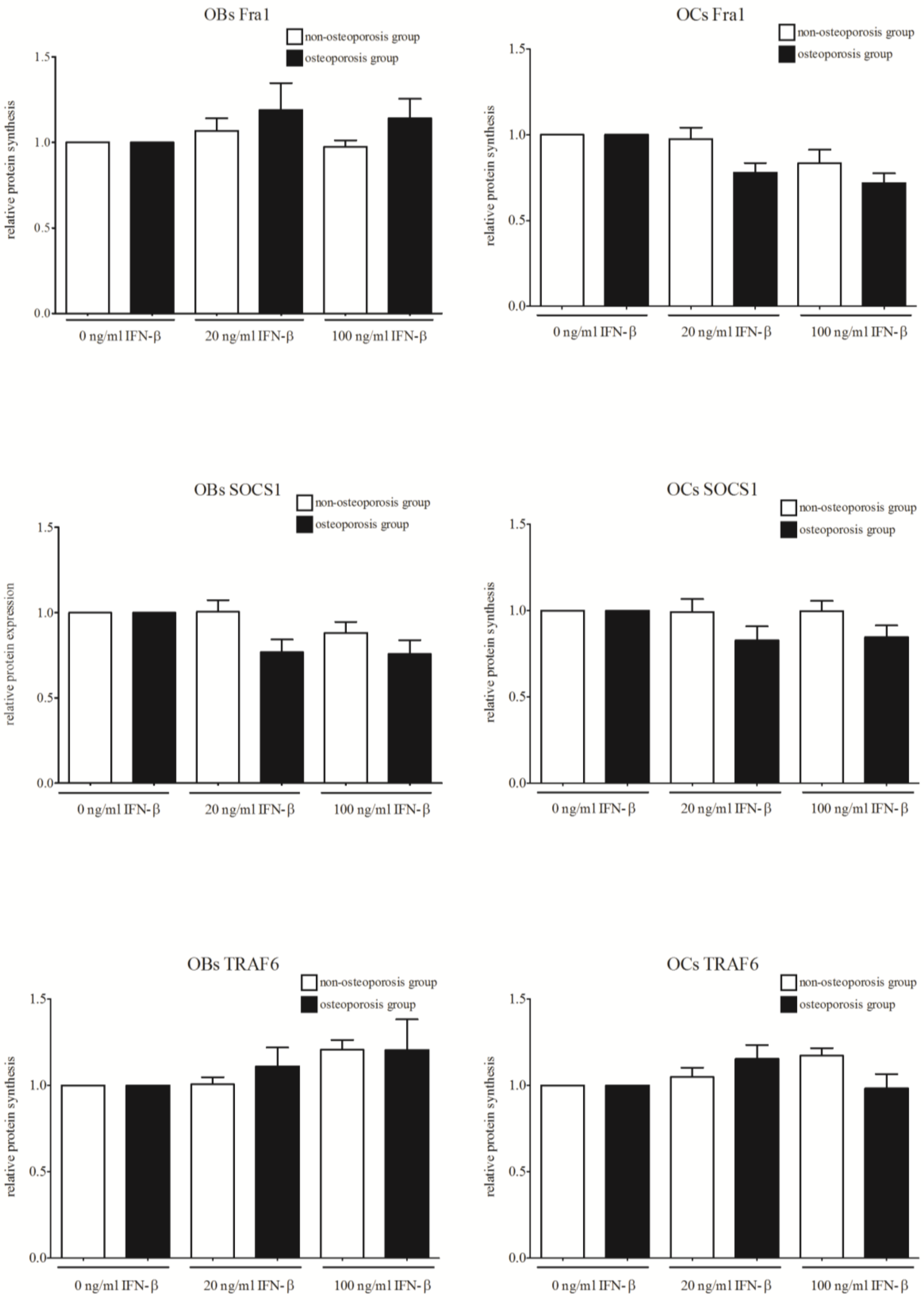

Figure AF4 - Protein levels of Fra1, SOCS1 and TRAF6 in osteoblasts and osteoclasts stimulated with IFN-β. fos-related antigen 1 (Fra1), suppressor of cytokine signaling 1 (SOCS1), TNF receptor-associated factor 6 (TRAF6), osteoblasts (OBs), osteoclasts (OCs)

Tables:

Table 1 - Demographical characteristics of the samples

	osteoporosis group	**non**-osteoporosis group
included patients (N)	12	11
Age (y), mean ± SD	78.7 ± 8.1	70.3 ± 10.4
Body mass index, ± SD	24.7 ± 2.2	28.0 ± 4.8
Gender (m:f)	4:8	6:5
main diagnosis (beside osteoporosis)	femoral neck fracture, coxarthrosis	femoral neck fracture, coxarthrosis
Bone density (mg/dl) ± SD	75.1 ± 12.3	217.2 ± 27.6

Table 2 - Primer sequences and PCR conditions

osteocalcin (OC), osteoprotegerin (OPG) and collagen1A1 (COL1A1), glyceraldehyde 3-phosphate dehydrogenase (GAPDH)

Name	Accession No.	Forward Primer 5' → 3'	Reverse Primer 5' → 3'	T_m (°*C*)	Size (bp)
OC	NM_199173.3	CCAGCGGTGCAGAGTCCAGC	GACACCCTAGACCGGGCCGT	60	236
OPG	NM_002546.3	CCGGAAACAGTGAATCAACTC	AGGTTAGCATGTCCAATGTG	60	313
COL1A1	NM_000088.3	AGCGGACGCTAACCCCCTCC	CAGACGGGACAGCACTCGCC	60	340
GAPDH	NM_002046.3	GTCAGTGGTGGACCTGACCT	AGGGGTCTACATGGCAACTG	60	420

Table 3 - Antibodies description and using conditions

fos-related antigen 1 (Fra1), suppressor of cytokine signaling 1 (SOCS1), signal transducer and activator of transcription 1 (STAT1), TNF receptor-associated factor 6 (TRAF6)

Name	Host species	Size (kDa)	Dilution	Company
β-actin	Mouse	43	1:1000	Merck Milipore
Fra1 (FOSL1)	Rabbit	38	1:1000	Sigma
SOCS1	Rabbit	38	1:1000	Sigma
p-STAT1	Rabbit	84/91	1:1000	Cell Signaling
STAT1	Rabbit	84/91	1:1000	Cell Signaling
TRAF6	Mouse	60	1:500	Santa Cruz
